# Hybrid approach for accurate water demand prediction using socio-economic and climatic factors with ELM optimization

**DOI:** 10.1016/j.heliyon.2024.e25028

**Published:** 2024-01-20

**Authors:** Zhaohui Li, Gang Wang, Danfeng Lin, Arsam Mashhadi

**Affiliations:** a School of Foreign Languages, Changchun University of Finance and Economics, Changchun, 130122, Jilin, China; b Shandong Transport Vocational College, Weifang, 261206, Shandong, China; c Tehran University, Tehran, Iran; d College of Technical Engineering, The Islamic University, Najaf, Iraq

**Keywords:** Water demand, Socio-economic variables, Climatic variables, The developed ant nesting algorithm, Elman neural network

## Abstract

This study proposes a hybrid approach for accurately predicting water demand by integrating socio-economic variables, such as population and GDP (per capita), with climatic variables, including temperature and precipitation. The prediction model utilizes an Extreme Learning Machine (ELM), effectively capturing the dynamic relationships between the input variables and water demand. The Improved Ant Nesting Algorithm is employed to fine-tune the weights and biases to optimize the network's performance. To evaluate the predictive accuracy of the model, a comprehensive dataset consisting of socio-economic and climatic factors is utilized for training and testing purposes. Performance metrics, namely Root Mean Square Error (RMSE) and Correlation Coefficients (R^2^), are employed as evaluation criteria. The results demonstrate that the hybrid approach achieves accurate water supply predictions, showcasing its potential to contribute significantly to effective water resource management and decision-making processes. Based on the results, IANA-ELM is considered the best model due to its high R^2^ values. Specifically, in the training data, the R^2^ values are 0.693 for population, 0.624 for GDP per capita, 0.607 for temperature, and 0.708 for rainfall. Similarly, in the test data, the R^2^ values are 0.672 for population, 0.608 for GDP per capita, 0.592 for temperature, and 0.708 for rainfall. This integrated approach provides a robust tool for policymakers, water utility companies, and researchers in the field of water managements, enabling them to make informed decisions based on accurate predictions of water demand.

## 1 Introduction

The importance of water in the contemporary world cannot be overstated, as it plays a crucial role in promoting socioeconomic progress and preserving the environment. Its significance is evident in various aspects of our lives, including agriculture, industry, energy production, and domestic use [1]. Furthermore, water is essential for maintaining ecological balance and preserving biodiversity, as it supports the survival of diverse plant and animal species. Therefore, ensuring adequate access to clean and safe water is crucial for sustainable development and the well-being of humans and planet well-being [2].

Water demand pertains to the amount of water persons need for different uses, such as residential, industrial, agricultural, public, and firefighting activities. Several variables, including population size, level of economic development, climatic conditions, lifestyle choices, and water accessibility, contribute to determining water demand. Quantifying water demand often entails assessing the quantity of water extracted or consumed from various sources, such as surface water, groundwater, or desalinated water. Water withdrawal is the quantity of water extracted from a particular source, while water consumption refers to the fraction not returned to the head after use. Furthermore, water demand may be quantified per capita, denoting the mean quantity of water used by each person within a specific region or nation [3].

Water demand is a critical indicator of both water scarcity and water management. Water stress occurs when the water demand exceeds the available supply or when water quality is impaired due to pollution or excessive use. Water administration is the systematic management, development, allocation, and oversight of water resources to meet the needs of both people and the natural world. The main goal of water management is to establish a balanced equilibrium between the demand and supply of water while also guaranteeing the sustainable and efficient use of water resources [4].

There is a multitude of factors that can impact the demand for water, including but not limited to the size of the population, the level of employment, the state of the economy, the advancements in technology, the effects of global warming, and the overall climate conditions. Understanding and analyzing these factors is crucial for predicting and managing water demand. Water is essential for domestic, municipal, industrial, agricultural, and environmental use [5]. Effective strategies are needed to manage this resource sustainably. Changes in technology and the environment can affect water supply and demand, requiring careful analysis. Global climate change may significantly impact regional water supply and demand, but it is challenging to predict the effects. Overall, managing water consumption is a complex matter that requires careful consideration and analysis [6].

Accurate and reliable water demand forecasts are crucial for effectively designing, operating, and managing urban water supply systems. These forecasts offer insights into different communities' expected water consumption patterns, enabling stakeholders to make informed decisions about infrastructure development and resource allocation [7]. Advanced data analytics and modeling techniques can be utilized to generate water demand forecasts with a high degree of precision, ensuring that urban water supply systems can meet the needs of growing populations while preserving natural resources [8]. Utilities must know the expected water demand for today and tomorrow to ensure that their treatment plants and wells can provide the appropriate amount of water [9]. They must also forecast water demand for 20–30 years to develop new water sources and expand their treatment plants [10]. To optimize their operational and investment decisions, utilities must accurately account for the uncertainty in demand forecasts, quantifying the risk of water shortages and revenue risk [11]. Demand forecasting is a crucial component of resource managements for both governments and private companies, especially given the scarcity of water and its inherent constraints. Water demand forecasts depend on various factors, such as the system's inertia, seasonality, type, and number of clients, as well as external factors that affect consumption. Short-term (daily) forecasting is particularly important for the efficient managements of water in storage tanks and related equipment [12].

Planning for decision-making forms the basis for forecasting in the water sector. To this end, a set of water demand forecasting literature differentiates forecast practice by the level of planning associated with the forecast or in accordance with the forecast horizon [13]. Regarding the planning level, all water demand forecasting exercises can be used for strategic, tactical, or operational decision-making. These concern capacity expansion, investment planning, system operation, managements, and optimization decisions. In terms of the forecast horizon, water demand forecasting can be categorized as either long-term, medium-term, or short-term, with these horizons reflective of the planning levels [14].

In the realm of water managements, effective decision-making and forecasting are critical components that must be considered. The water demand forecasting history outlines various forecast practices that differ based on the level of planning and forecast horizon [15]. Water demand forecasting serves as a vital tool for strategic, tactical, and operational decision-making, including but not limited to capacity expansion, investment planning, system operation, managements, and optimization [16]. Furthermore, water demand forecasting is classified based on the planning levels, namely long-term, medium-term, and short-term. The correct managements of water resources play a pivotal role in development as it facilitates poverty reduction and promotes fairness. Therefore, it is imperative to prioritize water managements and forecasting to ensure the sustainable use of this vital resource [17].

There has been a significant amount of research and experimentation conducted to develop effective models and methods for predicting water demand. This has involved exploring various approaches and techniques to better understand the factors influencing water usage and consumption patterns. For instance, various statistical approaches, linear and exponential combination models, and logarithmic models were constructed [18]. Through ongoing analysis and refinement, experts continue to work towards improving the accuracy and reliability of water demand prediction models, with the ultimate goal of supporting more effective water management strategies and decision-making processes [21]. In the next segment, we will delve into the investigations conducted by a range of researchers who have implemented diverse methodologies for forecasting water demand [19].

Rasifaghihi et al. [1] proposed a technique for predicting water usage through the utilization of Bayesian statistical approaches. The correlation between water usage and air temperature was used to divide observed daily water usage and climate variables into base and seasonal water use through clustering analysis. This study revealed that the fundamental water usage was not influenced by climate change but experienced fluctuations during the weekends. On the other hand, seasonal water usage was influenced by the daily temperature and overall precipitation. The researchers’ predictions factored in the uncertainties related to climate variables and model parameters. A probability distribution of daily water consumption was obtained through Bayesian linear regression. Multiple general circulation methods were utilized to acquire climate projections, which were then downscaled for Greater Montreal. The daily maximum temperature, minimum temperature, and total precipitation data were corrected for bias before being used as input for a Bayesian linear regression model to predict water consumption over the next thirty years. The results of the forecast indicated a consistent increase in seasonal water usage over time.

Three water demand forecasting models were developed by Guo et al. [2] by analyzing the utilization of water resources. These models included a logarithmic model, a linear and exponential combination model, and a hybrid model that combines linear, exponential, and logarithmic functions. A method for estimating water resource demand more accurately was suggested using a whale optimization algorithm incorporating social learning and wavelet mutation strategy. The algorithm introduced a linear incremental probability that enhanced the chances of a global search. The social network for an individual was created using social learning principles, incorporating social ranking and social influence. An adaptive neighborhood learning strategy was developed based on network relationships to enhance the sharing and exchanging of information among groups. By integrating the Morlet wavelet mutation mechanism, the algorithm was able to dynamically adjust the mutation space. This enhancement improved its ability to avoid local optimization. The proposed algorithm was confirmed to be superior in the latest benchmark functions, including CEC2017. An experiment was conducted using Shaanxi Province's water consumption data from 2004 to 2016. The outcomes indicated that the suggested algorithm was more effective than others in solving the three water resource forecasting models. The validity of the model was confirmed, and the practicality of the suggested algorithm was demonstrated with a prediction accuracy of 99.68 %.

Between 1980 and 2010, Zubaidi et al. [3] conducted a study to identify potential links between monthly climate parameters and municipal water usage. Their primary objective was to test the effectiveness of a combination of methods, Artificial Neural Networks (ANNs) and Singular Spectrum Analysis (SSA), in accurately forecasting long-term, monthly water demands. This was the first known study of its kind. It was discovered that Singular Spectrum Analysis proved to be effective in eliminating the effects of socio-economic factors and disturbances, as well as in identifying a random signal for long-term water consumption trends. Based on the findings, optimizing Artificial Neural Network using the Lightning Search Algorithm (LSA-ANN) resulted in better performance compared to other techniques, such as Gravitational Search Algorithm (GSA-ANN) and hybrid Particle Swarm Optimization (PSO-ANN). The suggested LSA-ANN methodology proved to be both accurate and strong. The correlation coefficient between predicted and observed water demand was 0.96 when a validation dataset was used, and the root mean square error was only 0.025.

Salloom et al. [4] proposed a solution to address errors that occur at certain points. This approach involved inserting virtual data within the actual data to minimize nonlinearity in those areas. As far as they knew, this was the first time anyone had addressed the issue of extreme points in this particular context. Additionally, the model for predicting water demand that they put forward was a Deep Learning model that did not require much complexity. To handle the sequential relationship in the historical demand data, the basic model utilized the Gated Recurrent Unit (GRU). Additionally, to improve the accuracy of forecasting with fewer parameters, an unsupervised classification method called k-means was implemented to create features. The suggested model was trained and tested using data from two water plants in China. By using this method, the complexity of the model was reduced by six times compared to previous literature while still maintaining the same level of accuracy. Additionally, the research discovered that expanding the data set had a significant impact on reducing errors by approximately 30 %. However, it did increase the duration of the training process.

Zubaidi et al. [5] presented a methodology that could be used to forecast monthly water demand. This method utilized a combination of approaches such as discrete wavelet transform, principal component analysis, particle swarm optimization, and several weather variable scenarios. Their methodology outperformed traditional methods in terms of both runtime and predictive accuracy. Data on water consumption and weather patterns in Melbourne City from 2006 to 2015 were generated by South East Water retail company. The findings indicated that pre-processing approaches could enhance the data quality and select the most suitable input scenario for the model. Moreover, the particle swarm optimization algorithm was observed to accurately forecast the constants of the suggested model. Additionally, the outcomes confirmed that the proposed methodology precisely estimated the monthly data of municipal water demand on the basis of various statistical criteria.

The importance of comprehending and forecasting water demand is apparent in the literature. After extensive investigation, it is apparent that numerous academics have made noteworthy advancements in their areas of expertise. Studies conducted on water demand forecasting have typically concentrated on climatic variables or socio-economic factors separately. However, there is still a need for an advanced model that combines climate change and socio-economic factors to optimize the accuracy and comprehensiveness of forecasting and decision-making in predicting water demand. As time passes, the importance of considering socio-economic and climatic variables in predicting water demand increases. Therefore, a new approach is required to predict water demand accurately and comprehensively.

The purpose of this study is to address the inadequacies of existing models for forecasting water demand and identify a more effective model for predicting water demand. The study employs a hybridized methodology that combines socio-economic and climatic variables to accurately and comprehensively predict water demand. To achieve this, the Elman neural network is optimized using the Developed Ant Nesting Algorithm, which effectively captures the dynamic relationships between input variables and water demand. Furthermore, a comprehensive dataset is utilized for training and evaluation, ensuring reliable performance assessment. This study's successful and precise predictions of water demand provide significant to effective water resource managements and decision-making. As such, it offers a valuable tool for policymakers, water utility companies, and researchers in the field of water management, enabling them to make informed decisions and effectively manage water resources.

## 2 Method and material

### 2.1 The case study description and methodology steps

Water scarcity in northern China, including Beijing, has worsened due to limited resources and unfavorable climate conditions and increase population. The Beijing located at 39° 54′ 50″ N, 116° 23′ 30″ E. The. The minimum temperature recorded during the specified period is 24.88 Cᶱ, the average temperature is 26.55 Cᶱ, and the maximum temperature is 28.73 Cᶱ. Additionally, the total precipitation has been reported as 229.41 mm. Beijing, experiences a dry climate with cold winters and hot summers, resulting in low average per capita water availability. This issue is primarily due to droughts, low rainfall, population growth, and rapid economic and social development. Beijing is the second most populous city in China after Shanghai and the political, cultural, and educational center of the country. Excessive extraction and use of underground water resources pose risks to future water availability. The increasing demands from agriculture and urbanization have led to an alarming escalation of water shortage and pollution issues. Concerns have been raised that water resources in northern China could be completely depleted by 2050 if not implemented. Comprehensive and sustainable solutions are needed to manage water scarcity in northern China, including efficient water management practices, conservation, alternative sources, and raising public awareness. The government and stakeholders must prioritize water resource management and implement effective policies to ensure a sustainable and sufficient water supply for the northern regions, supporting their long-term economic and social development. Data required to predict water demand include population, GDP per capita, temperature, and precipitation as input variables in the ELM model. The results of processing this data predict water demand, which is the output variable. The input data has been obtained from various sources for 2000 to 2023. The population is taken from the statistical Institute of China [8]. GDP per capita is obtained by statistical data provided in the Organization for Economic Co-operation and Development (OECD) [20].

GDP, temperature, precipitation, and water consumption are all factors to consider. The data spans the years 2000–2023 and includes 24 observations. The data is split into two subsets: the training set (16 observations from 2000 to 2015 and the testing set (8 observations from 2016 to 2023). The training set is used to train the ELM model, while the testing set is used to assess the model's accuracy and performance.

The sources of data are as follows:

- Population: The population data comes from the Chinese Statistical Institute, which offers yearly estimates of China's population size and growth rate.
- GDP per capita: The GDP per capita data is derived from the Organization for Economic Cooperation and Development (OECD) statistics data, which measures economic production per person in each nation.
- Temperature and Precipitation: The temperature data is collected from the National Meteorological Information Center of China, which gathers and analyses meteorological data from numerous stations around China.
- Water demand: The water demand data is generated using data from the Beijing Water Authority, which monitors and manages the city's water supply and demand.

The possible biases or restrictions connected with the dataset are as follows:

Because it only analyses a few parameters that impact water demand, such as population, GDP per capita, temperature, and precipitation, the dataset may not reflect the entire complexity and dynamics of the water shortage situation in northern China. Water price, water policy, water efficiency, water quality, water allocation, water reuse, and water culture may all impact water demand.

- Because it solely includes data from Beijing, one of the region's most water-scarce and water-stressed cities, the dataset may not accurately represent the regional and temporal variability of water demand throughout northern China. Water demand in various towns and provinces in the north of China may vary based on their geographic position, climatic conditions, economic growth, and sociological features.
- Because it solely utilizes historical data to train and test the ELM model, the dataset may not account for the volatility and unpredictability of water demand in northern China. Water demand may alter in the future due to climate change, population increase, urbanization, industry, and technological innovation.

**Fig. 1:**
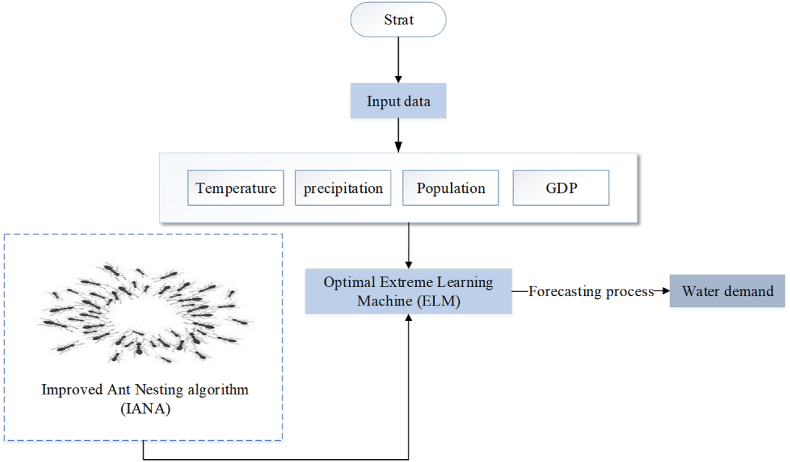
The methodology steps.

The methodology steps had been shown in Fig. (1).

### 2.2 Extreme learning machine (ELM)

**Fig. 2:**
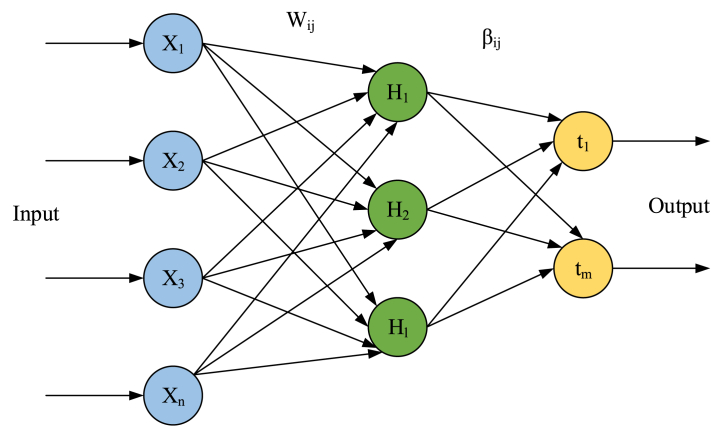
A sample configuration of the ELM network.

The Extreme Learning Machine model is one of the neural network models widely considered in recent years. It has many benefits, including its high speed in learning, easy usage, and capability to be utilized in numerous non-linear kernel functions and activation functions. The usage of an Extreme Learning Machine model can offer a combined format that includes several options for feature transmissions in the hidden layer. This format can be applied to regression and categorization tasks. This algorithm is a method for training SHNN (Single Hidden Neural Networks) that involves randomly initializing input biases and weights and evaluating output weights. The network can be trained quickly in a few steps using this approach. The arrangement of a basic Extreme Learning Machine network is illustrated in Fig. (2).

The equation (1) is for an arrangement with N number of training samples and D dimension [21].

(1) $$\left(x^{\left(n\right)},t^{\left(n\right)}\right),n=1:N$$

Here, $x^{\left(n\right)}\in R^{D}$ and $t^{\left(n\right)}\in R^{K}$.

The formulation of an Extreme Learning Machine-based feed-forward neural network can be as follows [equation (2)]:

(2) $$\sum_{m=1}^{M}\beta_{m}g\left(w_{m}^{T}x^{\left(n\right)}+b_{m}\right)=t^{\left(n\right)}$$

Here, the activation function is defined by g(.). The $m^{th}$ hidden neuron's bias is described by $b_{m}$. The hidden neurons are depicted by M. The input weight vector which joins the input neurons to the $m^{th}$ the hidden layer's neurons is demonstrated by $w_{m}=\left[w_{m1},w_{m2},\ldots ,w_{mD}\right]$. The weight vector which joins the $m^{th}$ neuron is determined by $\beta_{m}=\left[\beta_{m1},\beta_{m2},\ldots ,\beta_{mK}\right]$.

This concept is described as equation (3):

(3) Hβ=T

Where,

$$H={\left[\begin{array}{ccc} g\left(w_{m}^{T}x^{\left(1\right)}+b_{1}\right) & \cdots & g\left(w_{M}^{T}x^{\left(1\right)}+b_{1}\right)\\ \vdots & \ddots & \vdots \\ g\left(w_{1}^{T}x^{\left(N\right)}+b_{1}\right) & \ldots & g\left(w_{M}^{T}x^{\left(N\right)}+b_{M}\right) \end{array}\right]}_{N\times M}$$

$$H={\left[\beta_{1}^{T},\beta_{2}^{T},\ldots ,\beta_{M}^{T}\right]}_{M\times N}^{T};\;T={\left[t_{1}^{T},t_{2}^{T},\ldots ,t_{M}^{T}\right]}_{N\times K}^{T}$$

Here, a matrix that is non-square is demonstrated by H. The quantity of the hidden neurons is smaller than the quantity of the training samples. So, equation (4) is utilized in order to solve this problem:

(4) $$\hat{\beta }=H^{†}T$$

Here, the generalized Moore/Penrose matrix inverse is defined by $H^{†}$.

Therefore, the Extreme Learning Machine networks can be summarized into three primary stages:

First, random values are assigned to the input weights and biases. Then, the matrix of hidden neurons' outputs, matrix H, is evaluated. Finally, the weights matrix of the hidden neurons’ outputs is evaluated using Eq. (15).

### 2.3 Improved Ant Nesting Algorithm

To optimize the efficiency of the Extreme Learning Machine (ELM), the Improved Ant Nesting Algorithm (IANA) is used to precisely adjust the weights and biases of the hidden layer. The objective of this optimization method is to enhance the accuracy and prediction capabilities of the ELM.

The foraging and nest-building behavior of ants influence the IANA algorithm. The system replicates the innate actions of ants, such as using pheromone trails, and employs them to modify the weight and bias of the ELM's hidden layer. By using this approach, the algorithm enhances the efficiency of the ELM's learning process, allowing it to discern intricate patterns and connections within the provided data more effectively. The ELM can efficiently modify and optimize its parameters using the IANA algorithm, resulting in enhanced model performance and heightened precision in predicting water demand.

This section explains the Improved Ant Nesting Algorithm (IANA). The primary rationale for selecting this method is its capacity to efficiently explore the solution space and identify optimum or nearly optimal solutions. By repeatedly optimizing the network parameters using the technique, we may boost the performance of the ELM model and increase the accuracy of the water demand projections.

#### 2.3.1 Individual organisms

When the Leptothorax ant behaves as a simulation by creating a novel nest, the worker ants are regarded as the participants of the artificial foraging procedure. Whenever the Leptothorax ant acts as a simulation by making a new nest, the ants that are workers are considered candidates for the artificial foraging process. In a colony, a candidate of the artificial searching process could use every condition near the queen as a possible result. Workers drop grain into a colony, which they use. The global optimum result is demonstrated by the best condition to depose among all probable locations, which workers also utilize. Following this, workers’ condition indicates the condition for depositing.

In the Ant Nest Algorithm, we can describe the performance index as the depositing condition's explanation, its proximity to other stones, and how it affects the walls' organization. In a swarm, every worker's decision factor for depositing grain in a special condition is recognized as $d_{w}$ (deposition weight).

$d_{w}$ has shown how the worker's weight is randomly determined to deposit grain under specific conditions based on performance and outcome values for both the previous and current conditions. The details of this theory will be explained in the following section.

Regarding Ant Nest Optimization, the following points are crucial: First, workers search for agents. Second, the outcome of capacity is determined by depositing conditions. Third, the performance index is specified based on depositing conditions. Fourth, the workers’ decision element is the deposition weight ($d_{w}$). Fifth, the optimized outcome is achieved through the best depositing conditions. Finally, the previous depositing condition is determined by the stationary stone or nest mate.

When workers are searching for a new location to deposit grain, they may encounter stationary stones or other nest mates. To determine the presence of these obstacles, the previous deposit location of the worker $Z_{g,former}$, is taken into account, and the presence of the stationary stone/nest mate is explained in detail. Essentially, the location of the present worker's previous deposit indicates the stationary stone/nest mate they may encounter during the optimization process.

#### 2.3.2 Mathematical-based modeling

The suggested method is mainly driven by the worker ants' nesting behavior. It involves simulating the workers’ search for a suitable location among various options to deposit grain in this method. It is important to keep in mind that in the natural world, worker ants collect grains and transport them back to the nest. They follow a regular cycle to find a spot to deposit the grains and build a solid wall to protect the queen. The optimization method demonstrates only the process of dropping grains during nesting and imitates the behavior of the ants as they are searching for a suitable location to deposit a single grain. Initially, the approach did not consider the workers who built the first ones. The initial workers utilize the brood accumulation to automatically indicate the wall nesting location. Once the first deposition is simulated, the act of dropping grain occurs in this approach.

The initial method involves randomly placing artificial worker swarms across the solution $Z_{c}$ (i = 1, 2, 3, …, m) to improve the depositing process. Each worker's outcome represents a recently discovered depositing condition. Through random searching, the workers attempt to find a better location for depositing. The discovery of a superior deposition location is indicated by the optimal outcome. If a newly found location is inferior to the previous one, the search process continues until a better one is found.

Individuals in the natural world search for depositing locations in a random manner. In the proposed procedure, workers find the area with the weight of deposition by chance. Therefore, each time an individual discovers a new deposition location, which is represented by $Z_{g+1,c}$ (c = [1, m], g = [1, n]. Here, n shows the number of repetitions, and m represents the number of swarms. To recreate the deposition's location, equation (5) can be used:

(5) $$Z_{g+1,c}=Z_{g,c}+{\Delta Z}_{g+1,c}$$

Here, the repetition of the existent is demonstrated by c, and g stands for individual. The members' location of deposition is represented by Z, and the ratio change of deposition location is illustrated by Z and ${\Delta Z}_{g+1,c}$. ${\Delta Z}_{g+1,c}$ highly depends on $(d_{w}$), $Z_{g,c}$ (the present candidate), and $Z_{g,c,finset}$ (the difference among the best member's location of deposition). So, every member has the ability to improve the location where they deposit (probable result) by taking action towards becoming the best candidate (the best possible result). ${\Delta Z}_{g+1,c}$ is illustrated by equation (6):

(6) $${\Delta Z}_{g+1,c}=d_{w}\times \left(Z_{g,c,finset}-c\right)$$

The equation (7) is used for ${\Delta Z}_{g+1,c}$, if the member is the best:

(7) $${\Delta Z}_{g+1,c}=S\times \left(Z_{g,c}\right)$$

So, the present location is equal to the former one [equation (8)]:

(8) $${\Delta Z}_{g+1,c}=S\times \left(Z_{g,c,finset}-Z_{g,c}\right)$$

S stands for a number that is randomly selected, and it ranges between −1to1.

**Fig. 3:**
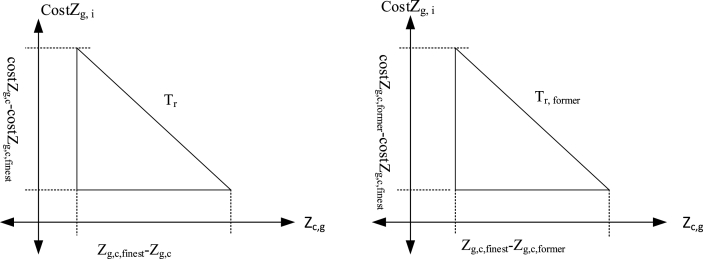
Commutating $T_{r}$ and $T_{r,former}$.

$d_{w}$ demonstrates the random walking of the members’ model on the basis of mathematics. This variable is dependent on the present and former depositing tendency ratio rate of workers ($T_{r}$ and $T_{r,former}$) at a special position for grain. In the Pythagorean equation, the sides of the slope illustrate $T_{r}$ and $T_{r,former}$ among the present and the former deposition location of the members. The technique of commutating $T_{r}$ and $T_{r,former}$ have illustrated in Fig. (3):

It has been computed for one candidate. At last, $d_{w}$ is represented in equation (9), and it is for minimizing the issue.

(9) $$d_{w}=R\times \left(T_{r}/T_{r,former}\right)$$

S is a random number, which ranges between [-1,1]. R takes a deposition role in order to have $d_{w}$ in control. In locations where consistent motion is desired, Levy flight is utilized solely to generate random quantities. It is worth mentioning that the distribution curve of the Levy flight is exceptionally excellent. The equations (10), (11) are utilized in order to calculate the previous and the current depositing tendency of workers ($T_{r,former}$ and $T_{r}$):

(10) $$T_{r}=\sqrt{{\left(Z_{g,c,finset}-Z_{g,c}\right)}^{2}-{\left(Cost\;Z_{g,c,finset}-{Cost\;Z}_{g,c}\right)}^{2}}$$

(11) $$T_{r,former}=\sqrt{{\left(Z_{g,c,finset}-Z_{g,c,former}\right)}^{2}-{\left(Cost\;Z_{g,c,finset}-{Cost\;Z}_{g,c,former}\right)}^{2}}$$

$Z_{g,c,former}$ illustrates the workers' previous deposition status. The performance index of the previous deposition status is represented by ${Cost\;Z}_{g,c,former}$, the performance index of the finest deposition status is demonstrated by $Cost\;Z_{g,c,finset}$ and the performance index of the existing worker is shown by ${Cost\;Z}_{g,c}$.

#### 2.3.3 Working mechanism

Regarding the highest and lowest boundaries, there are some random deposition statuses ($Z_{g,c}$ (c = [1, m], g = [1, n]) for the individuals of the swarm. Each candidate's previous deposition status ($Z_{g,c,former}$) is allocated to $Z_{g,c}$ at the outset due to the fact that it is the first generation. Then, $Z_{g,c,finset}$ is chosen for every epoch as the best deposition in the world. S is a random number, which ranges between [−1,1]. Furthermore, for each candidate, a comparison between $Z_{g,c,finset,}$ and $Z_{g,c}$ has been implemented. According to the formulation ${\Delta Y}_{t+1,i}=R\times (Y_{t,i}$), it can be deduced that the present worker of deposition status is the best outcome in the world, which means $Y_{t,i}=Y_{t,i,finset}$. However, According to the formulation ${\Delta Z}_{g+1,c}=S\times (Z_{g,c,finset}-Z_{g,c}$), it can be deduced that there are not any differences between the previous deposition status equal to current one. Else, ${\Delta Z}_{g+1,c}$, $d_{w}$, $T_{r,former}$, and $T_{r}$ must be determined according to the aforementioned formulations.

A new outcome was detected by the utilization of Eq. (9) in the following. Whenever the candidates detect a new outcome, a performance index should be performed to see whether the new outcome is better or not.

The previous outcome is labeled as $Z_{g,c,former}$, while the new discovery is adopted. Furthermore, if the current outcome is superior to the new one, it will be kept until the next iteration.

To optimize the results, implementing the suggested strategy would require two modifications to the equation. In the first step, $d_{w}$ has to be calculated with the utilization of equation (12):

(12) $$d_{w}=S\times \left(T_{r,former}/T_{r}\right)$$

The second modification must be represented in order to select the best outcome.

#### 2.3.4 Improved Ant Nesting Algorithm (IANA)

Modifying the Ant Nesting Algorithm is necessary to improve its ability to solve optimization problems. Any limitations or inefficiencies present in the original algorithm can be addressed through improvements. A modification that has been implemented in this case involves incorporating opposition-based learning (OBL) and the Sine map chaos theory into the algorithm.

##### 2.3.4.1 Opposition-based learning (OBL)

There is a strategy called Opposition-based learning that enhances the exploitation and exploration potentials of optimality algorithms. It represents the opposition's notion that includes taking the reverse of a solution in the space of the search into account. By the use of Opposite-Based Learning in the War Strategy optimization, the improved model is able to discover finer optima and explore an extensive variety of solutions.

The method produces a corresponding value for every individual. Considering a haphazardly chosen individual is involved in supplement value's computation, which has been illustrated as $Z_{g,c}^{C}$ in equation (13):

(13) $$Z_{g,c}^{C}={\bar{Z}}_{g,c}+{\underline{Z}}_{g,c}-Z_{g,c}$$

The opposite position of $Z_{g,c}$ has been denoted by the $Z_{g,c}^{C}$, while the upper and lower boundaries of the solution have been, in turn, illustrated by ${\bar{Z}}_{g,c}$ and ${\underline{Z}}_{g,c}$. Here, it can be seen that 40 % of the real candidates have been updated by the OBL. Improved exploration by the use of reverse solutions has been offered by the OBL, providing possibly finer and various solutions. Discovering various areas in the space of search concurrently can enhance convergence, which leads to the acceleration of the algorithm in the direction of an optimized solution.

##### 2.3.4.2 Sine Map Chaos Theory

This is a mathematical notion that represents a chaotic manner of optimizing algorithms. By adding the Sine map chaos theory to the improved Ant Nesting Algorithm, it takes advantage of chaotic properties such as random behavior and sensitivity to initial conditions. The algorithm utilizes Sine map chaos in the present position [equation (14), (15)].

(14) $${\Delta Z}_{g+1,c}=r_{i}\times \left(Z_{g,c,finset}-Z_{g,c}\right)$$

Where,

(15) $$r_{i+1}=\frac{\alpha }{4}\times \sin\left(\pi r_{i}\right)\text{,}$$

Here, $r_{0}=0.5$, and α=4 [22]

Some of the merits, like the enhanced search of the globe and improved variation, have been offered by Sine Map Chaos Theory. It assists the algorithm in discovering an extensive array of solutions and escaping local optima that leads to varied individuals. In addition, the ability of the algorithm to perform searches of the globe has been increased by the chaotic manner that enables finer solutions to be found away from the present individuals.

## 3 The results and discussion

The analysis of the test functions in the examined algorithms has utilized four indicators: minimum value, maximum value, average value, and standard deviation. These metrics offer significant insights into the performance and dependability of algorithms. The minimal value denotes the optimal outcome attained by an algorithm, signifying the utmost degree of precision and effectiveness. A maximum value signifies the top boundary of the algorithm's output. The average number is a comprehensive indicator of the algorithm's performance across multiple test functions. A lower mean value signifies superior optimization capabilities and shows the algorithm's constant capacity to attain high-quality outcomes. The standard deviation quantifies the extent of dispersion or variability exhibited by the algorithm's outputs. A smaller standard deviation indicates that the algorithm regularly generates outcomes that exhibit little variance from the mean value. This algorithm demonstrates enhanced stability and reliability. The process of the verification included the assessment of the Improved War Strategy Optimization on the CEC-BC-2017 exam suite functions. In doing so, the results of this algorithm were compared with five other algorithms, including Archimedes Optimization Algorithm (AOA) [23], Firefly Algorithm (FA) [24], Elephant Herding Optimization (EHO) [25], Wildebeest Herd Optimization (WHO) [26], and Lion Optimization Algorithm (LOA) [27]. Table 1 illustrates the efficiency suggested algorithm compered to examined algorithms.

**Table 1:** Comparative analysis of the proposed IANA method and other existing algorithms.

Function	indicator	LOA	WHO	EHO	FA	AOA	IANA
F1	Best	3.89	4.13	3.12	243.72	3.72	1.31
	Mean	24.36	27.15	30.18	412.82	27.08	5.44
	StD	9.96	21.88	27.34	242.59	10.91	10.63
F2	Best	6.25	6.66	5.68	6.09	6.09	0.09
	Mean	83.47	64.82	67.09	87.91	98.97	0.00
	StD	64.19	44.50	50.75	43.38	69.13	0.27
F3	Best	46.97	33.98	4.09	52.90	1.13	0.01
	Mean	54.48	65.85	10.65	49.61	1.15	0.02
	StD	7.85	7.88	23.83	21.64	1.12	0.00
F4	Best	8.63	7.66	6.09	6.89	1.28	0.06
	Mean	23.25	9.28	23.52	7.02	1.58	0.14
	StD	2.56	3.27	2.75	2.08	1.28	0.09
F5	Best	5.57	6.67	1.24	4.59	0.00	0.00
	Mean	5.62	6.09	2.73	5.61	1.12	0.01
	StD	2.64	2.60	1.93	2.99	0.00	0.00
F6	Best	1.27	1.22	2.87	1.27	0.00	0.00
	Mean	2.67	2.75	2.24	2.65	0.00	0.00
	StD	2.69	3.09	3.94	3.37	0.00	0.00
F7	Best	2.08	1.92	1.97	1.79	1.73	0.42
	Mean	3.05	2.43	2.65	2.36	3.09	0.81
	StD	1.42	1.44	1.31	1.41	1.34	0.12
F8	Best	24.85	22.78	24.74	10.23	21.47	4.18
	Mean	29.22	25.57	32.09	23.82	32.76	13.12
	StD	5.79	6.37	6.68	4.72	6.39	1.15
F9	Best	23.35	23.96	1.29	8.66	0.00	0.00
	Mean	36.30	38.82	3.28	44.65	0.00	0.00
	StD	24.79	26.05	2.81	32.40	0.00	0.00
F10	Best	68.69	97.19	3.99	6.38	1.24	0.20
	Mean	326.31	267.94	21.99	23.04	5.52	2.71
	StD	52.11	50.79	6.85	7.82	5.56	3.13
F11	Best	1.28	1.22	1.24	1.17	0.00	0.00
	Mean	1.27	1.25	1.58	1.33	1.12	0.00
	StD	1.21	1.23	1.19	1.17	0.00	0.00
F12	Best	0.00	0.00	0.00	0.00	0.00	0.00
	Mean	0.00	0.00	0.00	0.00	0.00	0.00
	StD	0.00	0.00	0.00	0.00	0.00	0.00
	StD	0.00	0.00	0.00	0.00	0.00	0.00

According to the outcomes of the four indicators above, it is evident that the proposed algorithm outperforms the other algorithms examined in this research. Suggested algorithm constantly attains minimal values for all test functions and showcases remarkable optimization skills and dependability. The consistent achievement of the lowest values of the suggested algorithm demonstrates its ability to recognize optimum solutions and provide exact outcomes.

Therefore, based on a thorough examination of these four indicators and their comparison with other algorithms, it can be inferred that the suggested method has exceptional performance, precision, efficiency, and notable applicability for problem domain.

Due to the algorithm's good performance in effectively managing optimization problems, the (IANA) has been used to optimize the Extreme Learning Machine (ELM) in this research. The choice to use this algorithm is based on its shown efficacy in successfully solving intricate optimization problems. Consequently, in our study, it is a helpful tool for improving the performance of the ELM.

Optimal extreme learning machine (ELM) used input variables of population, GDP per capita, temperature, and rainfall to predict water demand in this research.

The 28-year dataset has been recently used for model training and testing. In the training phase, 65 % of the total input data has been used to train the model. The remaining 35 % of the input data is saved for testing the performance of the trained model. Historical data from 2000 to 2015 has been used in the training process. This step allows the model to learn patterns and relationships within the data. After that, the trained ELM hybrid model is tested to simulat water demand from 2016 to 2023.

**Fig. 4:**
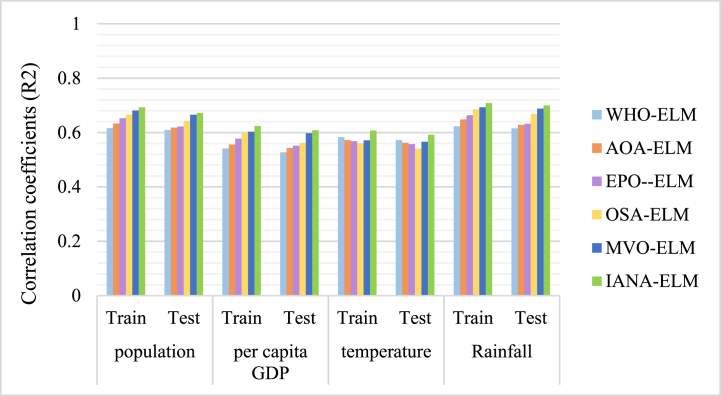
Correlation coefficients of combined ELM models by the input variables.

**Fig. 5:**
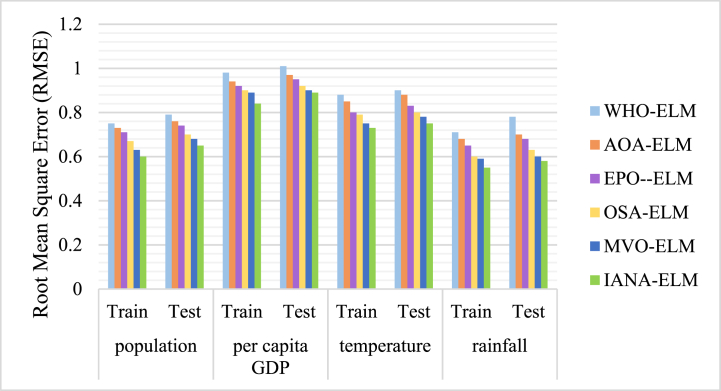
Root Mean Square Error (RMSE) of combined ELM models by the input variables.

The results obtained from the training and testing stages are compared with the actual values between 2000 and 2023. The results obtained from the model's performance by the input variables are presented in Fig. (4) and Fig. (5).

Fig. 4 presents a comparison of six models (WHO-ELM, AOA-ELM, EPO-ELM, OSA-ELM, MVO-ELM, and IANA-ELM) that use Extreme Learning Machines (ELM) and various optimization techniques for water demand forecasting. The performance is evaluated using R2 values, which quantify the degree of fit between the model and the data (with 1 indicating a perfect match and 0 meaning no fit). In summary:

• WHO-ELM: the poorest performance, poorest R^2^ values (0.527–0.623 testing, 0.541–0.616 training), minimum accuracy, maximum bias, underfits data.
• AOA-ELM: moderate improvement, marginally better R^2^ values (0.543–0.648 testing, 0.556–0.633 training), slight accuracy, slight bias, sufficiently fits data.
• EPO-ELM: improved performance, improved R2 values (0.551–0.663 testing, 0.577–0.653 training), improved accuracy, low bias, and improved data fit.
• OSA-ELM: good efficiency, good R2 values (0.541–0.685 testing, 0.56–0.666 training), good accuracy, low bias, fits data good.
• MVO-ELM: better performance, better R2 values (0.566–0.693 testing, 0.571–0.681 training), better accuracy, low bias, fits data very better.
• IANA-ELM: best performance, maximum R2 values (0.592–0.708 testing, 0.607–0.693 training), best accuracy, low bias, fits data perfectly.
• The RMSE values of six combined models are shown in Fig. (5). The RMSE values demonstrate the model's deviation from the actual data; lower is better. The synopsis is as follows:
• WHO-ELM: poorest fit, highest RMSE values (0.79–1.01 testing, 0.75–0.98 training), poorest accuracy, maximum error.
• AOA-ELM: moderate improvement, moderate lower RMSE values (0.76–0.97 testing, 0.73–0.94 training), moderate accuracy, slight error.
• EPO-ELM: improved fit, improved RMSE values (0.74–0.95 testing, 0.71–0.92 training), improved accuracy, improved error.
• OSA-ELM: good fit, low RMSE values (0.7–0.92 testing, 0.67–0.9 training), good accuracy, low error.
• MVO-ELM: better fit, lower RMSE values (0.68–0.9 testing, 0.63–0.89 training), better accuracy, lower error.
• IANA-ELM: best fit, lowest RMSE values (0.65–0.89 testing, 0.6–0.84 training), maximum accuracy, minimum error.

The study reveals that IANA-ELM has the highest correlation coefficient and lowest error values in predicting the relationships between input variables during the training and testing phases. This indicates its excellent performance in accurately forecasting outcomes, with minimal discrepancies between anticipated and observed data. Population and rainfall showed the most robust association coefficients during training and testing, indicating their significant impact on future water demand. Population expansion and a reduction in precipitation are expected to affect future water demands significantly.

**Fig. 6:**
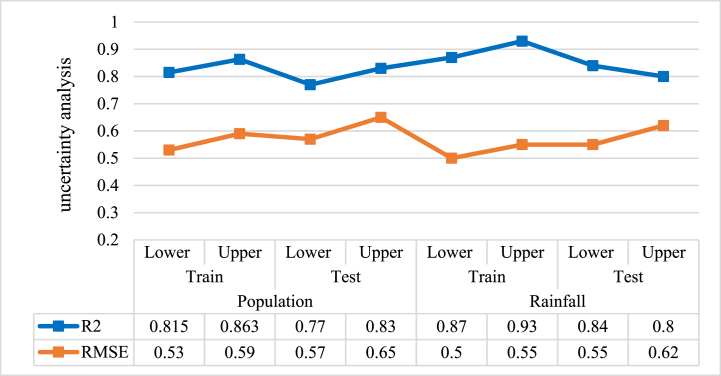
The correlation and error value of the uncertainty analysis.

Due to the critical importance of population and precipitation parameters, an uncertainty analysis has been performed to evaluate the accuracy of the prediction of these variables. Fig. (6) shows the correlation and error measures obtained from the uncertainty analysis of population and rainfall forecasts during both training and testing phases.

According to the results, the model fits the data well and has high accuracy, as shown by high R2 (0.77–0.93) and low RMSE (0.5–0.65) for both steps. The results show that precipitation substantially affects water demand more than population, so the highest R2 and the lowest RMSE are for rainfall.

**Fig. 7:**
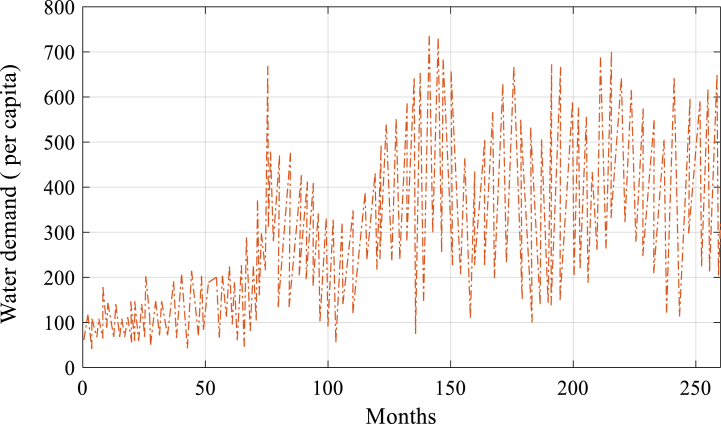
The simulated value combined suggested model.

**Fig. 8:**
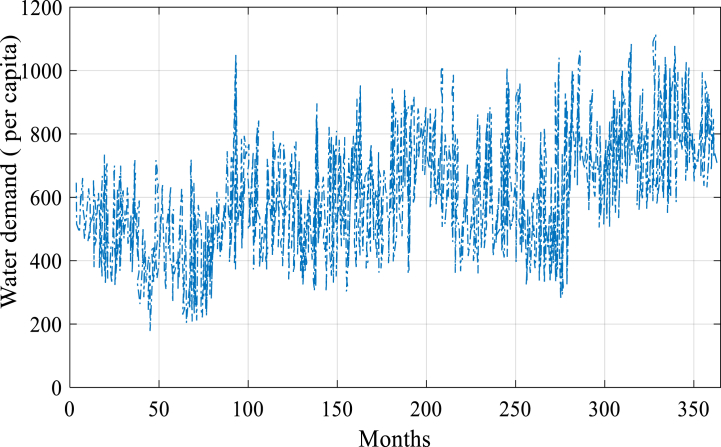
The forecasted value combined suggested model.

After successfully implementing the proposed model, it predicts water demand from 2023 to 2050, considering population, GDP per capita, precipitation, and temperature. By combining these variables, the model provides accurate predictions of water demand. The model facilitates strategic decision-making and helps achieve the predicted value, as shown in Fig. (7) and Fig (8).

The simulation and the water demand forecast are the two main components of the model outputs. While the projection (2023–2050) demonstrates how the model predicts future trends, the simulation (2000–2022) illustrates how effectively the model replicates the previous data. The simulated and predicted values are shown in Fig. 7, Fig. 8, respectively.

Fig. (7) demonstrates a tight correspondence between the simulated values and the actual data, as they exhibit a consistent pattern and direction. The suggested enhanced algorithm in the combined ELM technique is responsible for achieving high accuracy. This algorithm efficiently resolves optimization concerns and improves the model's learning capability. The high correlation between the simulated and observed numbers provides further evidence for the correctness of the simulation findings.

Fig. (8) illustrates the model's water demand prediction from 2023 to 2050. This projection is based on the anticipated values of independent variables, including population, per capita GDP, temperature, and rainfall. Fig. 8 depicts a rising trajectory in water demand in the future, indicating the projected increase in population and economic development, as well as the potential impacts of climate change.

The findings from the second part emphasize that the city of Beijing will experience a continuous upward trajectory in water demand due to the combined effects of population growth and changing climatic conditions. Consequently, there may be limitations in meeting the future water requirements of the country. Furthermore, the accurate forecasting of electricity demand empowers managers to make informed decisions regarding resource allocation and proper planning to avoid potential electricity supply shortages in society.

It is crucial to compare the recommended technique with existing water demand forecasting methods to analyze the proposed hybrid strategy thoroughly. This study may provide a more comprehensive comprehension of the advantages and disadvantages of the suggested methodology. Table 2 presents a comprehensive comparison study of the proposed hybrid strategy and its investigation.

**Table 2:** Comparative analysis between the proposed hybrid approach and other model.

Study	Model	Accuracy	RMSE
[28]	MLR	0.67	1.98
[29]	ANFIS	0.81	1.38
[30]	LSTM	0.85	1.02
	CNN-LSTM	0.88	0.91

The effectiveness of the proposed method is obvious when analyzing the results shown in Fig. (4) to Fig. (6). The accuracy of the model and RMSE compared to those published in previous research projects show the improvement of the performance and reliability of the suggested model in forecasting water demand.

The study's results indicate that the hybrid technique can effectively apply to various data sets and geographic locations. The fundamental ideas and methods of the hybrid system, which integrate statistical modeling with machine learning approaches, are not intrinsically linked to a particular dataset or geographical area. This implies that the procedure may be readily adjusted and implemented in many circumstances.

Nevertheless, it is crucial to recognize that certain restrictions and boundaries could exist while using the hybrid strategy in various situations. The hybrid system's performance may be affected by differences in data availability, quality, and features in multiple areas. For instance, variations in data availability or the efficacy of data-gathering methods across different regions might impact the technique's suitability.

Furthermore, it is necessary to verify and adjust the performance of the hybrid strategy while implementing it in different situations to guarantee its efficacy. The predicted accuracy of the mixed technique may be affected by various environmental, social, or economic aspects in other geographic locations. Considering these elements when implementing the process in new contexts is essential.

To summaries, while the suggested hybrid strategy displays potential for being applicable in many situations, it is crucial to acknowledge the possible limits and restrictions of its implementation. Conducting more validation tests and comparison analyses in other geographic locations and data situations would provide valuable insights into the adaptability and efficacy of the hybrid strategy. This will facilitate the comprehension of academics and practitioners on the most effective way to use the approach in various circumstances and pinpoint areas needing more improvement or customization.

The precise forecasts produced by our hybrid methodology may provide useful insights for policymakers in making well-informed choices on water resource management. Here is an explanation of how these forecasts might aid in identifying regions with substantial water needs and distributing resources accordingly:

- Locating Areas with High Water Demand: Policymakers may acquire precise water demand projections for various regions or areas within a city or region by analyzing historical data and using our hybrid technique. These projections may identify areas that regularly have elevated water demand trends. This data enables policymakers to pinpoint hotspots or areas experiencing substantial water scarcity, necessitating targeted actions or conservation measures.
- Providing Resources Correctly: Planners can distribute water resources more effectively and efficiently by making precise forecasts of water demand. Investments in infrastructure development, such as increasing water delivery networks or enhancing storage facilities, might be prioritized in regions that are expected to have high future water demand. By adopting a proactive strategy, the aim is to guarantee enough water supply that can effectively cater to the needs of the people, hence minimizing the likelihood of water scarcity or insufficient distribution.
- Planning Conserving strategies: Precise forecasts allow policymakers to develop and execute focused conservation plans. To prioritize water conservation efforts, governments target regions with significant water use and pursue measures such as supporting effective irrigation methods, advocating for water-efficient technology, and increasing public knowledge on responsible water utilization. These conservation initiatives may effectively mitigate the total water use and lessen the burden on current water supplies.
- Long-Term Planning: The hybrid approach's accurate forecasts may also help policymakers with long-term infrastructure planning. Policymakers may make educated judgments about building or upgrading water infrastructure to suit future demands by studying expected water demand trends. In locations where water demand is predicted to expand dramatically, this involves building additional reservoirs, developing water reuse systems, and investing in desalination facilities.

## 4 Conclusion

This study presents a hybrid approach incorporating socioeconomic and climatic variables using an Extreme Learning Machine (ELM) and an improved Ant Nesting Algorithm. The model accurately predicts water demand by considering population, GDP per capita, temperature, and precipitation factors. The fine-tuning weights and biases through the Improved Ant Nesting Algorithm further optimize the prediction model's performance. The simulation results from 2000 to 2023 show high accuracy, closely aligning with real values. The improved algorithm addresses optimization problems in ELM method. The model's projections for electricity demand from 2023 to 2050 show an upward trajectory, emphasizing the challenges policymakers and water resource managers face in meeting future water demands, especially in cities like Beijing. By leveraging accurate water demand predictions, decision-makers can develop proactive strategies for effective water resource management. This integrated approach offers a robust tool for policymakers, water utility companies, and researchers in water management, enabling well-informed decisions based on accurate water demand forecasts. The paper suggests that further research be done on water consumption, with a focus on forecasting beyond 2050 in order to grasp long-term trends and related challenges fully. The suggested hybrid method suggests further area research to establish water management policies. The proposed hybrid technique uses socioeconomic and climatic data, which may differ across places and countries. Because data sources and preparation processes impact the model's accuracy and generalizability, it is advised that data from the researched areas be used. A sensitivity analysis would also be performed to assess the model's robustness and to identify the key elements that impact changes in water demand.

## Data availability statement

Research data are not shared.

## Additional information

No additional information is available for this paper.

## CRediT authorship contribution statement

**Zhaohui Li:** Formal analysis, Data curation, Conceptualization. **Gang Wang:** Methodology, Investigation. **Danfeng Lin:** Formal analysis, Data curation, Conceptualization. **Arsam Mashhadi:** Writing – review & editing, Writing – original draft, Formal analysis, Conceptualization.

## Declaration of competing interest

The authors declare that they have no known competing financial interests or personal relationships that could have appeared to influence the work reported in this paper.
